# All You Need Is Light. Photorepair of UV-Induced Pyrimidine Dimers

**DOI:** 10.3390/genes11111304

**Published:** 2020-11-04

**Authors:** Agnieszka Katarzyna Banaś, Piotr Zgłobicki, Ewa Kowalska, Aneta Bażant, Dariusz Dziga, Wojciech Strzałka

**Affiliations:** 1 Department of Plant Biotechnology, Faculty of Biochemistry, Biophysics and Biotechnology, Jagiellonian University, Gronostajowa 7, 30-387 Krakow, Poland; a_katarzyna.banas@uj.edu.pl (A.K.B.); piotr.zglobicki@uj.edu.pl (P.Z.); ewa.b.kowalska@uj.edu.pl (E.K.); aneta.bazant@doctoral.uj.edu.pl (A.B.); 2 Department of Microbiology, Faculty of Biochemistry, Biophysics and Biotechnology, Jagiellonian University, Gronostajowa 7, 30-387 Krakow, Poland; dariusz.dziga@uj.edu.pl

**Keywords:** Cryptochrome-*Drosophila*, *Arabidopsis*, *Synechocystis*, human proteins (CRY-DASH), cyclobutane pyrimidine dimer (CPD), DNA damage, DNA repair, photolyase, photoreactivation, pyrimidine dimer, ultraviolet, (6-4) pyrimidine–pyrimidone photoproduct (6-4 PP)

## Abstract

Although solar light is indispensable for the functioning of plants, this environmental factor may also cause damage to living cells. Apart from the visible range, including wavelengths used in photosynthesis, the ultraviolet (UV) light present in solar irradiation reaches the Earth’s surface. The high energy of UV causes damage to many cellular components, with DNA as one of the targets. Putting together the puzzle-like elements responsible for the repair of UV-induced DNA damage is of special importance in understanding how plants ensure the stability of their genomes between generations. In this review, we have presented the information on DNA damage produced under UV with a special focus on the pyrimidine dimers formed between the neighboring pyrimidines in a DNA strand. These dimers are highly mutagenic and cytotoxic, thus their repair is essential for the maintenance of suitable genetic information. In prokaryotic and eukaryotic cells, with the exception of placental mammals, this is achieved by means of highly efficient photorepair, dependent on blue/UVA light, which is performed by specialized enzymes known as photolyases. Photolyase properties, as well as their structure, specificity and action mechanism, have been briefly discussed in this paper. Additionally, the main gaps in our knowledge on the functioning of light repair in plant organelles, its regulation and its interaction between different DNA repair systems in plants have been highlighted.

## 1. Introduction

**Figure 1:**
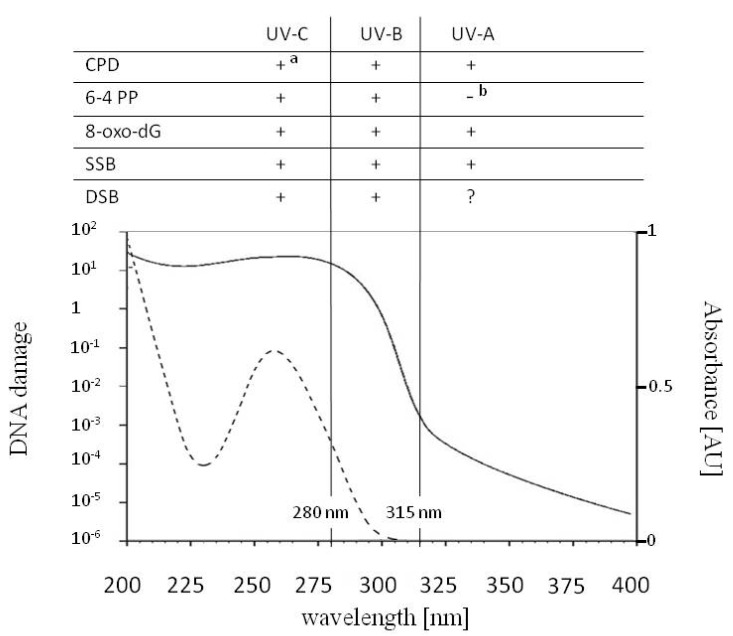
The common DNA lesions induced by UV-A (315–400 nm), UV-B (280–315 nm) and UV-C (100–280 nm), the action spectrum for generalized DNA damage from 200–400 nm (solid line) and the absorption spectrum for DNA with the maximum at 260 nm (dotted line). Cyclobutane pyrimidine dimers (CPDs), 7,8-dihydro-2′-deoxyguanosine (8-oxo-dG) and single strand breaks (SSBs) are formed upon UV-A, UV-B and UV-C radiation. CPDs can undergo photoreversion to undamaged bases upon UV-C radiation (a). UV-B and UV-C are responsible for the formation of (6-4) pyrimidine–pyrimidone photoproducts (6-4 PPs) and double strand breaks (DSBs). The role of UV-A in the direct formation of DSBs is controversial. While under a biologically relevant intensity of UV-A formation of 6-4 PPs does not occur, upon absorption of UV-A, 6-4 PPs can isomerize to Dewar isomers (b). Under UV-A, isolated 6-4 PPs can act as photosensitizers leading, to production of CPDs and SSBs.

About 10% of the electromagnetic energy emitted by the Sun is between 100 and 400 nm [1]. At the Second International Congress on Light in Copenhagen in 1932, William Coblentz suggested that these wavelengths be subdivided into UV-A, UV-B and UV-C. As recommended by the Comité International del’Éclairage (CIE), the range of UV-A is from 315 to 400 nm, UV-B is from 280 to 315 nm, and UV-C is from 100 to 280 nm [2,3,4] (Figure 1). Before reaching the uppermost layer of the Earth’s atmosphere, UV-A, UV-B and UV-C constitute 6.8, 2.4 and 0.8% of the solar radiation energy, respectively [5]. UV absorption by the stratospheric ozone reaches its maximum at about 260 nm [6]. Together with Rayleigh scattering, it limits the amount of UV radiation at the Earth’s surface, with UV-C becoming completely absorbed [1]. As a consequence, around 5.7 and 0.3% of sunlight energy at sea level is in the UV-A and UV-B range, respectively [7], but their ratio depends on several factors, including latitude, altitude, day of the year, time of day and clouding. UV-B is the part of solar radiation with the shortest wavelengths, i.e., the highest energy that reaches the Earth’s surface.

UV may be absorbed directly by many constituents of living cells, which affects their functioning or causes damage indirectly due to the production of reactive oxygen species (ROS). UV absorption by aromatic amino acid residues may generate free thiol groups when an excited-state electron is transferred to a nearby disulfide bridge. Free thiol groups may, in turn, react with each other and form new disulfide bridges [8]. Besides the induction of a disulfide bridge, the ROS produced upon UV absorption may mediate other amino acid modifications, including glutathionylation, nitrosylation and carbonylation. The above processes may cause conformational changes, influencing protein functions and activities as well as protein–protein interactions. Furthermore, UV induces the formation of covalent bonds between nucleic acids and the neighboring proteins. The UV-dependent crosslinking between RNA and ribosomal proteins was detected in both maize cytoplasmic and chloroplast ribosomes [9]. The accumulation of these RNA–protein crosslinks may inhibit transiently the translation process in maize leaves. The ROS generated by UV can also affect lipids with double C-C bonds of polyunsaturated fatty acids as their main targets. The lipid-forming membranes and those which are components of signaling cascades may likely be damaged by peroxidation. The peroxyl and alkoxy radicals generated in this cascade process may further affect the neighboring molecules. Additionally, apart from proteins and lipids, nucleic acids are also the targets of UV radiation. Damage to DNA may be cytotoxic or mutagenic, and it impairs the functioning of living organisms, eventually leading to their death. 

Since life emerged on Earth, all the organisms have had to cope with high UV intensity. The levels of UV-A, UV-B and UV-C emitted by the Sun were higher when the Earth was young than they are at present; furthermore, the two latter bands were not absorbed by the ozone layer then, as it had not formed yet [10]. Thus, living organisms, especially sessile ones which use solar light as the energy source, had to evolve mechanisms allowing them to deal with UV-induced DNA damage. These mechanisms include a reduction in the UV dose reaching the cell by avoiding UV exposure, the production of UV-absorbing compounds or the repair of damage which could not be avoided.

## 2. UV Impact on DNA

Each nucleotide in nucleic acid absorbs in a UV range with two peaks, the first at about 200 nm, the second ranging from 254 to 271 nm depending on the nucleotide [11]. Thus, DNA of different origins, including *Escherichia coli* native DNA, isolated plasmid and adenoviral DNA, typically displays two absorption peaks at about 200 nm and 260 nm [12,13,14] (Figure 1). The fluorescence quantum yield of individual nucleotides is extremely small as a result of the very short lifetimes of their excited singlet states. The excited state lifetimes of single or double DNA strands are longer, which depends, among other things, on the content of the deoxyguanosine and nucleotide sequence determining the secondary, tertiary and quaternary structure of DNA [15]. Therefore, an excess of energy may be transferred between neighboring bases in a DNA strand, which is the case in the hypochromic effect, i.e., increased UV absorption upon denaturation of the DNA helix [16]. Despite the ultrafast deactivation of the electronic excited states of DNA bases, the remaining long-lived ones are responsible for the formation of DNA lesions [17].

The action spectrum, i.e., the overall biological effectiveness at each wavelength for generalized DNA damage in the range of 250 to 370 nm drawn up by Setlow [18], compiles several biological effects, e.g., the survival of *E. coli* or T4/T6 phages and the levels of different types of DNA lesions. Cocknell [10] expanded the action spectrum of DNA damage from 200 nm, proposing an almost flat shape in the 200–260 nm range, where the curve reaches its maximum and then falls at wavelengths above 260 nm (Figure 1). Due to this curve, the level of DNA damage induced by 260 nm (UV-C) is about 10^6^ times higher compared with irradiation at 360 nm (UV-A). 

**Figure 2:**
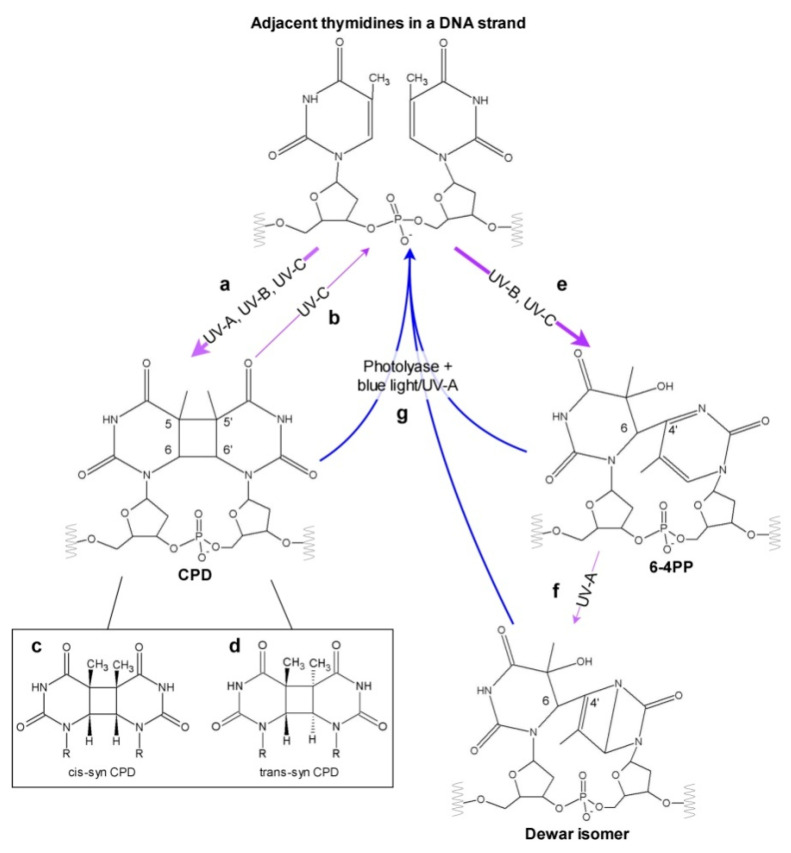
Diagram showing possible effects of different UV types on neighboring pyrimidines in a DNA strand. Absorption of UV-A, UV-B, UV-C may cause the formation of CPD (**a**), which can be split back by UV-C (**b**). Depending on the conformation of the two adjacent bases at the time of irradiation, different CPD stereoisomers can occur—mainly cis-syn CPD (**c**) and less frequently trans-syn CPD (**d**). 6-4 PPs are created upon absorption of UV in the UV-B or UV-C range (**e**), and can subsequently isomerize to Dewar photoproducts in UV-A (**f**). All three types of photoproducts can be repaired by photoreactivation performed by photolyases requiring blue or UV-A light (**g**), or by dark repair mechanisms (not shown). Thick and thin purple arrows mark the prevalent and less frequent processes, respectively.

The direct absorption of UV by DNA leads mainly to the formation of pyrimidine dimers between adjacent pyrimidines in a DNA strand. The most frequent photolesions are cyclobutane pyrimidine dimers (CPDs), followed by (6-4) pyrimidine–pyrimidone photoproducts (6-4 PPs) [19,20,21] (Figure 1 and Figure 2). 

In CPDs, a cyclobutane ring is formed between the C5 and C6 atoms of mainly TpT, and less frequently in TpC and CpC dipyrimidines (Figure 2) [21,22,23,24]. The proportion of CPDs formed at the above-mentioned pyrimidine–pyrimidine sites depends on the wavelength and DNA origin. This estimation depends also on the quantification method used. Twelve isomeric forms of CPDs have been described, however steric hindrance in duplex DNA in the B-form allows for the formation of cis-trans CPD isomers only [25,26]. CPDs absorb in a UV-C range with a maximum at 230 nm. Under low doses of UV-C, the production of CPDs is linear, and with increasing radiation intensity an equilibrium is observed between CPD formation and splitting [27]. As mentioned above, the whole radiation in the UV-C range is absorbed by the ozone layer. Thus, in natural sunlight, with UV-B as the shortest wavelength, the UV-C-dependent photoreversion of CPDs into undamaged bases does not occur. 

The second most common pyrimidine dimers are 6-4 PPs with a single covalent C4–C6 bond between the neighboring bases (Figure 2). They are produced most frequently at TpC sites and, in decreasing order, at the TpT, CpT and CpC sites [21,28]. Similarly to CPDs, the proportion of 6-4 PPs produced at the above-mentioned pyrimidine–pyrimidine sites varies between DNA of different origins and UV wavelengths. The estimation of this proportion is also influenced by the quantification method used. 6-4 PPs absorb in a UV-A range with a maximum between 310–330 nm, depending on the DNA sequence [29]. Upon absorption of UV-A photons, 6-4 PPs can undergo photoisomerization into Dewar valence isomers [30] (Figure 1 and Figure 2). Surprisingly, this isomerization may occur only in a DNA strand with an intact phosphate backbone, which is a key player in the process [31]. The presence of Dewar isomers was reported in etiolated cucumber cotyledons irradiated with UV-B followed by UV-A, but not in *Arabidopsis* plants or in suspension cultures in the same conditions [20,32]. 

Apart from direct absorption by DNA, UV can affect nucleic acids indirectly. It has been shown that CPDs may be induced in isolated DNA through a triplet–triplet energy transfer from UV-A-absorbing photosensitizers, including porphyrins, flavins, steroids and quinones [33]. Under in vitro conditions as well, isolated 6-4 PPs (but not ones located inside single-stranded or double-stranded DNA) may react with the neighboring molecules, leading to the induction of either CPDs or single-strand breaks (SSBs) [5,34]. It has been demonstrated that melanin may act in vivo as a photosensitizer [35]. The authors proposed that the ROS and nitrogen reactive species raised upon UV-A exposure are responsible for the excitation of electrons from melanin monomers. Such monomers can transfer the energy to DNA, leading to its damage. Thus, CPDs can be produced in murine skin in the darkness, even over 3 h after exposure to UV-A. However, there is no evidence of the involvement of native photosensitizers in the formation of pyrimidine dimers in plants exposed to UV. 

In addition to pyrimidine dimers, other DNA lesions may also be induced under UV (Figure 1). Such damage is not the effect of direct UV absorption by DNA, but results mainly from the generation of ROS. Oxidative stress may lead to, among other things, the production of 8-oxo-7,8-dihydro-2′-deoxyguanosine (8-oxo-dG) and 2,6-diamino-4-hydroxy-5-formamido- pyrimidine (Fapy) [19,36]. It has been shown that 8-oxo-dG may be induced by UV-A, UV-B and UV-C in plant cells [37,38]. 8-oxo-dG can be generated in a DNA helix or in a pool of free nucleotides, and incorporated into a DNA strand during replication. Finally, UV exposure may lead to the formation of strand breaks in a DNA helix. Experiments conducted in the 1990s showed that both SSBs and double strand breaks (DSBs) can be induced in animal cells after either UV-A or UV-B irradiation [39]. However, the role of UV-A in the formation of DSBs has been cast in doubt by recent research [40,41]. Greinert et al. [42] proposed that the DSBs detected in UV-A irradiated DNA originate from clusters of other oxidative DNA lesions. 

CPDs were found in genomic DNA irradiated with wavelengths between 330 and 261 nm (i.e., under UV-A, UV-B and UV-C) [43] (Figure 1). For the detectable induction of 6-4 PPs, irradiation with biologically relevant intensities of wavelengths no longer than 305 nm (i.e., with UV-B or UV-C) was needed [43,44]. The isomerization of 6-4 PPs into Dewar isomers in etiolated cucumber cotyledons reached the maximum when irradiated with 325 nm [32]. The amount of pyrimidine dimers and 8-oxo-dG rises with a decreasing wavelength, i.e., with an increase in photon energy [37,43,45,46,47]. Although CPDs are the most frequent UV-induced DNA lesions, the CPD to 6-4 PP ratio depends on several factors, including the UV wavelength, the DNA sequence and the investigated organism. The CPD to 6-4 PP ratio was 9:1 and 2:1 in an *Arabidopsis* suspension culture and in etiolated hypocotyls, respectively [20,48]. Douki et al. [44] showed that the intensity of UV-A present in a solar light produced CPDs, oxidized purines (mainly 8-oxo-dG) and produced SSBs in mammalian cells in a 10:3:1 ratio. 

The formation of pyrimidine dimers in isolated DNA decreases with the rising temperature, and is consistent with the DNA melting curve, i.e., the unbinding of two strands in a DNA duplex into single strands [49]. Therefore, it is hypothesized that the photoreactivity of single-stranded DNA is lower compared to double-stranded DNA, with a more prominent effect at higher temperatures. The impact of temperature on the induction of photolesions was also found in cellular DNA with the opposite effect. The amounts of both UV-induced CPDs and 6-4 PPs in etiolated cucumber cotyledons, a suspension culture of tobacco cells and *Arabidopsis* leaves do not decrease, and actually grow with a rising temperature in the range 0–25 °C [50,51,52]. This indicates a role of cell components, including proteins associated with DNA, in the temperature-dependent modulation of the generation of DNA lesions upon UV in cells. This may be due to UV absorption or energy transfer between the excited-state molecule, acting as a photosensitizer, and cellular DNA. 

## 3. Photoreactivation 

UV-induced lesions affect DNA metabolism and functions. The presence of pyrimidine dimers causes a distortion both in naked and nucleosomal DNA helixes [53,54,55,56]. Such steric hindrance blocks the progression of DNA and RNA polymerases, affecting cell functioning due to the disturbance in replication and transcription [57,58]. Moreover, the spontaneous deamination of cytosine in CPDs results in the formation of uracil derivatives, which are complemented with adenine during replication. This explains why CPDs are more mutagenic than 6-4 PPs [59].

It has been known since the late 1940s that the viability of bacteria cells increases when UV-exposure is followed by visible light [60]. This phenomenon results mainly from the activity of photoreactive enzymes called photolyases [61]. Photolyases were found in most investigated organisms. Placental mammals, as well as some bacteria, fungi, protozoans and nematodes, lost all their genes encoding functional photolyases in the course of evolution [62]. It was proven using atomic force microscopy that photolyases are associated with undamaged DNA, and that they slide through the strand to scan for pyrimidine dimers [63]. The affinity of photolyase to DNA with pyrimidine dimers is higher than to non-damaged DNA, and may differ as much as 7500 times, as shown for *E. coli* CPD-specific photolyase [64]. The binding of photolyase to pyrimidine dimers does not depend on light and occurs even in darkness [65]. An interaction between pyrimidine dimers and the binding pockets of photolyases allows these lesions to be flipped out from the DNA helix, which results in increased DNA flexibility [66,67,68]. CPDs and 6-4 PPs do not match with the same binding pocket of photolyases, because of various three dimensional structures and different efficiencies of hydrogen bond formation with interacting partners (Figure 2). Therefore, photolyases are specific to either CPDs or 6-4 PPs. However, recently a bifunctional photolyase capable of repairing both CPDs and 6-4 PPs has been described [69]. This enzyme, cloned from a UV-resistant bacterium *Sphingomonas* sp. UV9, has a unique binding pocket which fits both types of pyrimidine dimers.

In addition to the repair of 6-4 PPs, TpC- but not TpT-derived Dewar lesions can be repaired by 6-4 PP photolyase isolated from *Drosophila melanogaster* [70]. This is possible due to the structural similarities of 6-4 PPs and their Dewar derivatives (Figure 2). Interestingly, the substitution of distinct amino acids in the binding site of *Xenopus leavis* 6-4 PP photolyase causes modulation of its activity, which allows the repair of CPDs. An attempt to convert an *E. coli* CPD photolyase into the one repairing 6-4 PPs resulted in a non-functional protein [71]. 

Plant photolyases belong to three classes: (i) class II, specific to CPD, (ii) 6-4 PP-specific, and (iii) Cryptochrome-*Drosophila*, *Arabidopsis*, *Synechocystis*, Human (CRY-DASH) proteins/class 0 [72]. CRY-DASH/class 0 photolyases are responsible for CPD repairs in ssDNA [73,74]. Pokorny et al. [75] found that *Arabidopsis* CRY-DASH/class 0 protein encoded by *AtCRY3* can also bind and repair CPDs in dsDNA, but only when these lesions have been localized in a loop structure. The reason is the inability of Atcry3 to stabilize CPDs which have flipped out from the DNA duplex [73,75]. Other classes than the CRY-DASH/class 0 photolyases can operate on both ssDNA and dsDNA. 

**Figure 3:**
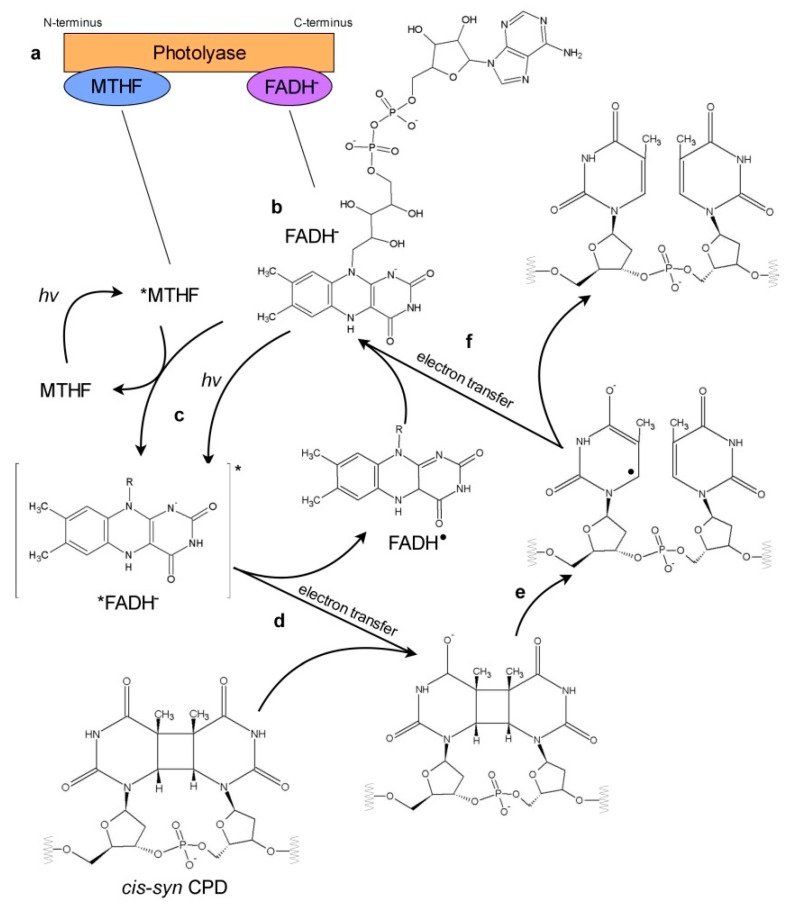
Schematic representation of electron transfer in photoreactivation. Known photolyases have two chromophores: non-covalently bound antenna chromophore (most commonly MTHF) near the N-terminus, and flavine adenine dinucleotide (FAD) near the C-terminus (**a**). FAD serves as a catalytic cofactor in the photoreactivation in the form of anionic hydroquinone (FADH^−^) (**b**). The other FAD states are represented in the simplified form with the group attached to the N-10 of the flavin moiety marked with R. The photorepair of CPD is shown as a representative model: (**c**) FADH^−^ is excited directly upon absorption of blue/UV-A photon or by energy transfer from excited *MTHF antenna chromophore. (**d**) Electron is transferred from excited *FADH^−^ onto a pyrimidine dimer. In this step, FADH^•^ radical and CPD^•^ radical are created. (**e**) Bonds between adjacent pyrimidines in a dimer are broken. Two separate rings are formed. (**f**) Electron is transferred back to FADH^•^ radical and finally undamaged pyrimidines and FADH^−^ are restored.

Photolyases have two non-covalently bound chromophores, flavin adenine dinucleotide (FAD), involved in splitting the pyrimidine dimer, and the second one acting as an antenna. FADH^−^ is the only redox state of FAD acting in photoreactivation. An excited-state electron from FADH^−^, which is a result of either the direct absorption of the blue/UV-A or an energy transfer from the antenna chromophore, is used in sequential steps to break the bonds between adjacent pyrimidines forming dimers. Thus, a simple reversion of pyrimidine dimers to individual pyrimidines occurs (Figure 3).

Among five redox states of FAD, two redox pairs, an oxidized flavin/anionic semiquinone (FAD/FAD^•−^) and a neutral semiquinone/anionic hydroquinone (FADH^•^/FADH^−^), exist in photolyases. FAD bound to photolyases has an unusual bent U-shaped structure, which determines the intramolecular energy transfer dynamics between its flavin and adenine moiety. Femtosecond spectroscopy studies revealed that among the four mentioned above FAD redox states only bound to photolyase FADH^−^ displays intramolecular energy transfer slower in the chromophore cofactor (i.e., flavin) and faster with the pyrimidine dimer substrate [76]. This may explain why only FADH^−^ serves as a catalytic cofactor in the photoreactivation.

Five different antenna chromophores have been identified so far, as follows: (i) 5,10-methenyltetrahydrofolate (MTHF), (ii) 8-hydroxydeazaflavin (8-HDF), (iii) 6,7-dimethyl-8-ribityl-lumazin (DMRL), (iv) FAD and (v) flavin mononucleotide (FMN) [77]. MTHF and HDF are the most common. DMRL was identified in some bacterial 6-4 PPs-specific photolyases, which are phylogenetically unrelated to the eukaryotic ones [78]. These enzymes have a sulfur–iron cluster similar to the one found in primases responsible for the synthesis of the RNA primer during DNA replication. 

Whereas the absorption spectrum of isolated FADH^−^ typically displays a peak around 325 nm (near-UV), it has a peak at 440 nm (blue light), or maxima ranging from 370 to 420 nm (UV-A to blue light), for photolyases with HDF or MTFH antenna chromophores, respectively [29]. Thus, although the presence of the antenna chromophore is not indispensable for photoreactivation, it extends the absorption cross-section of the photolyase and enhances its repair efficiency. 

As the antenna chromophore is loosely bound to a photolyase, it is often lost in protein purification. The absorption spectra of isolated FADH^−^, as well as the *Arabidopsis* and rice CPD photolyases expressed in *E. coli*, are similar [79,80,81]. The absorption spectrum of *Arabidopsis* CPD photolyase did not change even when the putative chromophore, MTHF, was added to the purified protein [79]. This indicates that MTHF does not serve as an antenna. The structural analysis of class II photolyases of higher plants confirms the lack of residues responsible for MTHF binding [77]. The action spectra of CPD photorepair in *Arabidopsis*, sorghum and cucumber, with a broad peak ranging from around 375 to 425 nm, as well as the absorption spectrum of the native rice CPD photolyase and *Arabidopsis* 6-4 PP photolyase, suggest that FMN or FAD can act as antennae in plant photolyases [32,81,82,83,84]. However, to date the only experimentally confirmed plant antenna chromophore is MTHF bound to Atcry3 isolated from *Arabidopsis* leaves [85].

The 0.8–0.9 efficiency of CPD repair is achieved by the prolonged life-time of the excited-state of FADH^−^ chromophore and the architecture of the photolyase active site, which allows for the stabilization of the charge-separated intermediate (FADH^•^ + CPD^•^) [76]. During the photoreactivation of CPDs, only electron transfer occurs, whereas the repair of 6-4 PPs requires both electron and proton transfer [76]. The proton transfer is a limiting step resulting in a much lower reaction efficiency of 0.1. Besides the 6-4 PPs splitting models, which assume the involvement of a transient oxetane-type structure, a transient formation of the water molecule was also proposed [86]. The whole photocycles of CPD and 6-4 PP repair take about 1.07 and 10.7 ns, respectively [76]. These differences between the CPD and 6-4 PP repair mechanisms, with the latter being more complex, explain why the conversion of CPD photolyase into an enzyme-repairing 6-4 PPs requires structural changes both in the binding pocket and in the whole protein structure [71].

Photolyases and blue light photoreceptor cryptochromes (crys) belong to one protein family [29]. They share the photolyase homology region (PHR), which binds both FAD and the antenna chromophore. In addition, plant and animal cryptochromes possess a C-terminal extension of variable length. This extension (absent in photolyases) is involved in cryptochrome signaling and its interaction with downstream components. Plant cryptochromes evolved from CPD photolyases, but they neither bind nor repair DNA lesions [87]. Nevertheless, the substitution of one amino acid in *Arabidopsis* cry1 has produced a protein which could repair CPDs in dsDNA, at least under in vitro conditions [88]. In this mutein, a fully reduced FADH^−^ typical for photolyases, not a half reduced FADH typical for cryptochromes, was accumulated upon blue light illumination. This provides further evidence confirming the key role of FADH^−^ in the splitting of the pyrimidine dimers [88,89].

Photoreactivation is the main mechanism used to repair pyrimidine dimers in non-proliferating plant cells [82,90,91]. As CPDs are not only the main UV-induced DNA lesions but are also the most mutagenic, their repair is pivotal for plant functioning. It is not surprising that the activity of CPD-specific photolyases apparently impacts not only plant survival under UV, but also the mutation rate, thus appearing to act as one of the players in plant evolution [92,93,94]. 

Despite a simple and very specific photoreactivation mechanism, it is insufficient to provide the proper maintenance of the genome integrity of plants exposed to UV. It is ineffective under weak light or at night following UV exposure. Photoreactivation cannot repair damage other than that to pyrimidine dimers formed in DNA, e.g., 8-oxoG, SSBs and DSBs. To cope with these problems, plants use other mechanisms independent of light repair. These are called dark repair, and the most prominent one is the nucleotide excision repair (NER). Details of the dark repair of UV-induced DNA damage in plants are described elsewhere [95,96].

## 4. Perspectives

The mode of action of most of the components responsible for DNA repair in animal and yeast cells has been comprehensively described, whereas there are still numerous gaps in the cognition of their plant homologue functioning. In this paper, we have discussed just a few issues which are awaiting elucidation. 

### 4.1. DNA Packaging, Metabolism and Interaction between Light and Dark Repair

Most data concerning the molecular mechanism of photoreactivation come from in vitro studies using isolated proteins and naked DNA. The issue of how photolyases act on native chromatin has not been frequently investigated. The flipping out of a pyrimidine dimer from a DNA helix is crucial for its repair by photolyase. One may expect that the photorepair efficiency should be influenced by the flexibility of DNA and the accessibility of the lesion. Actually, similarly to other repair pathways, photoreactivation operates preferentially in non-nucleosomal regions [97,98]. Experiments using yeast minichromosomes have revealed that, whereas the complete removal of CPDs in linker DNA and nuclease-sensitive regions takes 15–30 min, in the case of nucleosomal DNA, about 2 h are required [98]. UV irradiation induces the histone acetylation and DNA methylation observed in maize and *Arabidopsis,* respectively [99,100]. These modifications are responsible for chromatin remodeling, which in turn influences light and dark repair due to changes in DNA packaging. The interplay between chromatin structure, pyrimidine dimers and their repair is knotty. The levels of CPDs and 6-4 PPs influence DNA methylation in *Arabidopsis*, which presumably leads to chromatin rearrangements [100]. Whether and to what extent such rearrangements lead to the better accessibility of photolyases to pyrimidine dimers, and eventually to more efficient photorepair, needs to be elucidated. Interestingly, in yeast, photorepair was found to be faster in a non-transcribed strand [98,101]. This was interpreted as the blockage of access of photolyase to pyrimidine dimers by RNA polymerase II that stalled at the lesions. According to this interpretation, the repair of CPDs and 6-4 PPs in the course of transcription depends on the synchronized activity of the photolyase operating on a non-transcribed strand and that (NER) operating on a transcribed one [98,101]. 

The chromatin remodeling 8 (AtCHR8) protein may constitute the link between photolyases, RNA polymerase II and NER in plants. AtCHR8 is a homologue of human Cockayne’s syndrome group B (CSB) protein which is a part of a transcription-coupled excision repair pathway. CSB is responsible for the removal of RNA polymerase II stalled at the DNA lesions, and has a SWI/SNF domain responsible for ATP-dependent chromatin remodeling [102,103]. Recently, Khateeb et al. [104] have shown that UV impairs root growth in the *Arabidopsis chr8* mutant kept after irradiation either in light or in dark. Consequently, the authors proposed the involvement of AtCHR8 in light and dark repair. 

The strong binding of photolyases to pyrimidine dimers is independent of light, but these enzymes require light energy to repair the lesions. This generates the question of whether the access of other proteins involved in DNA repair to pyrimidine dimers may be blocked in darkness by bound photolyases, or alternatively, whether the photolyases bound to pyrimidine dimers can recruit other repairing proteins. The results of experiments conducted in the 1980s confirm the latter possibility. The expression of yeast CPD photolyase enhanced the survival of the UV-irradiated *Saccharomyces cerevisiae* cells lacking RAD18 protein involved in DSB repair. This effect was observed in neither the yeast *rad2* mutant of endonuclease involved in NER nor in *rad2rad18* cells. This points to the specific role of CPD photolyase in complementation of *rad18* mutation in yeasts [105]. Moreover, the presence of CPD photolyase influenced the NER repair of DNA lesions other than pyrimidine dimers, with opposite effects in *E. coli* and yeasts [106,107]. Whereas the expression of CPD photolyase decreased the survival of yeast exposed to cisplatin, it increased *E. coli* resistance to this drug. This increased resistance was proposed to be an effect of the enhanced excision repair of the cisplatin-l,2-d(GpG) adduct by *E. coli* photolyase, as proven in vitro. Fox et al. [106] found that yeast CPD photolyase can bind also to DNA lesions induced by several mutagens, including cis-platin, N-methyl-N’-nitro-N-nitroso-guanidine (*MNNG*), 4-nitroquinoline 1-oxide (*4NQO*) and nitrogen mustard (*HN2*), but does not repair them. Despite these intriguing findings, research was discontinued. 

Ataxia telangiectasia-mutated (ATM), Ataxia telangiectasia-mutated and Rad3-related (ATR) are crucial kinases responsible for the coordination of the eukaryotic cell responses to DNA damage. These serine/threonine kinases initiate signaling cascades leading to DNA repair. ATM and ATR are known to be activated by DSBs and SSBs, respectively [108]. Homologues of mammal/yeast ATM and ATR kinases were found in *Arabidopsis.* They were shown to have similar functions as in other organisms. Many physiological responses may be initiated following DNA damage. However, data are scarce on the signaling cascades triggered specifically by the UV-induced pyrimidine dimers in plants. AtATM and AtATR redundantly activate the UV-B induced programmed cell death of stem-cells in *Arabidopsis* roots [109]. It is been shown that this is connected with the cooperation between AtATM, AtATR and the translesion DNA polymerases stalled at the photoproducts [110]. AtATM and AtATR kinases are involved in the CPD-dependent inhibition of hypocotyl growth in etiolated *Arabidopsis* seedlings [48]. It was demonstrated that this inhibition was a consequence of the cell cycle arrest initiated by pyrimidine dimer accumulation. Only recently has it been shown that 6-4 PPs, but not CPDs, activate ATR signaling in human fibroblasts [111]. The level of pyrimidine dimers is modulated by the rate of their UV-dependent formation and the activity of photolyases. Whether there are links between plant cell signaling and the levels of pyrimidine dimers, whether CPDs activate different signaling pathways than 6-4 PPs in plants, whether ATM or ATR kinase is specifically activated in plants by pyrimidine dimers, and whether ATM or ATR can regulate photolyase functioning as a result of their phosphorylation—all these questions are awaiting elucidation.

### 4.2. Repair of UV-Induced Lesions in Chloroplasts 

An interplay between chloroplast functioning and the amount of mRNA of *Arabidopsis* 6-4 PP photolyase has been shown [112]. The disturbance of photosynthesis leads to an increased transcript level of this enzyme. Putative retrograde signaling is modulated by coaction between red light photoreceptors phytochrome A and phytochrome B. The evidence of photorepair in chloroplasts is confusing. Whereas *Arabidopsis* CPD photolyase is found only in the nucleus, rice CPD photolyase and *Arabidopsis* 6-4 PP photolyase are localized in the nuclei, chloroplast and mitochondria [93,112,113]. In addition, AtCRY3, a CRY-DASH/class 0 photolyase which can bind and repair CPDs localized in the ssDNA, is present in the chloroplast and mitochondria, but not in the nuclei [114]. However, the physiological role of AtCRY3 has not been established yet. Experiments performed in the 1990s confirmed photoreactivation in plant nuclei, but the photorepair of CPDs and 6-4 PPs in chloroplasts was observed neither in *Arabidopsis* seedlings nor in spinach leaves [84,115,116]. On the other hand, the light-dependence of the removal of unspecified DNA lesions from chloroplast DNA, and of the removal of CPDs and 6-4 PPs from the total DNA encompassing 25% of chloroplast DNA, was interpreted as proof of the efficient photorepair of pyrimidine dimers in chloroplasts of soybean suspension cells and *Arabidopsis* leaves, respectively [117,118]. Taking the data into account, this interpretation seems to be overestimated. Clear evidence of the photoreactivation of CPDs in rice chloroplasts and mitochondria was provided only by Takahashi et al. [113]. The enhancement of pyrimidine dimer repair in plants overexpressing photolyases suggests that the amount of these enzymes is a limiting factor [92,93,119]. The correlation between CPD photolyase expression and the level of photorepair enhances this assumption [120,121,122]. Thus, the precise distribution of a limited amount of photolyase molecules between the nuclei, chloroplasts and mitochondria, linked with the level of DNA damage in each organelle, seems to be of crucial importance for plant survival in a high UV environment. It may be assumed that post-translational modifications regulate this distribution.

### 4.3. The Role of Phosphorylation in Photorepair

It is worth noticing that photoreactivation is a very efficient repair mechanism, with the same protein responsible for the detection of the DNA lesion and its repair independently on the template strand. The other repairing pathways are multistep processes, and require the orchestrated actions of many proteins. The strict regulation of protein functions, subcellular localization and enzyme activities is possible due, among other factors, to the post-translational modifications that involve phosphorylation. Although there is only indirect evidence of the role of phosphorylation in the regulation of the repair of UV-damaged DNA in plants, the details that correlate these processes are little known. Phosphorylation of a serine localized in close proximity to a FAD chromophore in rice CPD photolyase was confirmed by mass spectrometry [81,123]. It was proposed that the phosphorylation of a residue localized in close proximity to a FAD chromophore may lead to conformational changes, influencing substrate binding and increasing the photorepair efficiency [124]. The above-described phosphorylated serine is located in the phosphate binding motif. Structural analysis shows the presence of such a motif in the PHR of rice CPD and in *Arabidopsis* 6-4 PP photolyases [123,124]. Thus, it cannot be excluded that both CPD- and 6-4PP-specific photolyases undergo phosphorylation. To date, only CPD photolyases from rice and wheat were demonstrated to be phosphorylated *in planta* [81,113,119]. As the phosphorylation status of rice CPD photolyase differs between organelles, its role in targeting photolyase into nuclei, chloroplasts and mitochondria was proposed [113]. Another possibility is that the phosphorylation or other post-transcriptional modifications are responsible for the regulation of photolyase activity, its DNA binding properties and its putative interaction with other proteins. The physiological role of photolyase phosphorylation, environmental factors regulating this process, and the involvement of kinases (ATM/ATR?)/phosphatases, are the other issues that require experimental verification.

## Abbreviations

ATM	Ataxia telangiectasia-mutated
ATR	Ataxia telangiectasia-mutated and Rad3-related
CHR8	chromatin remodeling 8
CPD	cyclobutane pyrimidine dimer
CRY	cryptochrome
CRY-DASH	Cryptochrome-*Drosophila*, *Arabidopsis*, *Synechocystis*, Human proteins
DMRL	6,7-dimethyl-8-ribityl-lumazin
DSB	double strand breaks
FAD	flavin adenine dinucleotide
MTHF	5,10-methenyltetrahydrofolate
NER	nucleotide excision repair
PHR	photolyase homology region
ROS	reactive oxygen species
SSB	single strand break
UV	ultraviolet
6-4 PP	(6-4) pyrimidine–pyrimidone photoproduct
8-HDF	8-hydroxydeazaflavin
